# Potential risk of re-emergence of urban transmission of Yellow Fever virus in Brazil facilitated by competent *Aedes* populations

**DOI:** 10.1038/s41598-017-05186-3

**Published:** 2017-07-07

**Authors:** Dinair Couto-Lima, Yoann Madec, Maria Ignez Bersot, Stephanie Silva Campos, Monique de Albuquerque Motta, Flávia Barreto dos Santos, Marie Vazeille, Pedro Fernando da Costa Vasconcelos, Ricardo Lourenço-de-Oliveira, Anna-Bella Failloux

**Affiliations:** 1 Instituto Oswaldo Cruz - FIOCRUZ, Rio de Janeiro, Brazil; 2 Institut Pasteur, Arboviruses and Insect Vectors, Paris, France; 3 Institut Pasteur, Epidemiology of infectious diseases, Paris, France; 4 000 0004 0620 4442 Instituto Evandro Chagas, Belém, Brazil

## Abstract

Yellow fever virus (YFV) causing a deadly viral disease is transmitted by the bite of infected mosquitoes. In Brazil, YFV is restricted to a forest cycle maintained between non-human primates and forest-canopy mosquitoes, where humans can be tangentially infected. Since late 2016, a growing number of human cases have been reported in Southeastern Brazil at the gates of the most populated areas of South America, the Atlantic coast, with Rio de Janeiro state hosting nearly 16 million people. We showed that the anthropophilic mosquitoes *Aedes aegypti* and *Aedes albopictus* as well as the YFV-enzootic mosquitoes *Haemagogus leucocelaenus* and *Sabethes albiprivus* from the YFV-free region of the Atlantic coast were highly susceptible to American and African YFV strains. Therefore, the risk of reemergence of urban YFV epidemics in South America is major with a virus introduced either from a forest cycle or by a traveler returning from the YFV-endemic region of Africa.

## Introduction

Yellow fever (YF) is an arboviral disease endemic to tropical regions of Africa and South America that may cause hemorrhagic fever in humans. The etiologic agent is the yellow fever virus (YFV), the prototype member of the genus *Flavivirus* (family Flaviviridae). It is a single-stranded, positive sense RNA virus with a genome of approximately 11 kb. Until now, seven lineages have been identified: five in Africa, with two in West Africa (West Africa I and II) and three in East and Central Africa (East Africa, East/Central Africa, and Angola), and two in the Americas (South America I and II)^1–3^. The YFV strains circulating in the Americas derived from a Western African lineage ancestor, and most isolates from Brazil belong to the South American genotype I^4, 5^. These findings are in line with the hypothesis that YFV emerged in Africa and was imported into the American East coast from West Africa during the slave trade which has undoubtedly favored the introduction of the African YF mosquito *Aedes* (*Stegomyia*) *aegypti* (Linnaeus)^2, 4^.

Despite the availability of effective vaccines, YF remains an important public health issue in Africa and South America, with an annual incidence of around 200,000 cases and 30,000 deaths^6^. The 2015–2016 YFV epidemic in Angola exemplifies the threat posed by the reemergence of this virus; a six-month urban outbreak in Angola reported 4,000 suspected cases and ~600 deaths. The epidemic reached the Democratic Republic of Congo, and viremic people dispersed to densely populated and *Aedes-*infected zones, in and outside Africa (e.g. China)^7^.

Yellow fever is a zoonotic disease with two main epidemiological transmission cycles: the forest and the urban cycles. Traditionally, the YFV is transmitted within a forest cycle between non-human primates (NHPs) and canopy-breeding mosquitoes. During epizootics of YF, humans can be contaminated if they live close to the forest fringe or when entering into the wild. While YFV in Africa circulates within a forest cycle before emergence in an urban cycle involving humans and *Ae. aegypti*, it has remained an enzootic virus restricted to rainforests and savannah-like forests in the Americas since the 1940s^3, 8^.

In the Americas, the disease was a major human health issue from the 18^th^ to the early 20^th^ century with devastating urban epidemics. However, the development of two attenuated vaccines in the 1930s as well as a continental eradication program of *Ae. aegypti* led to a clearance of urban YF^3^. Brazil was certified free of *Ae. aegypti* in 1957^9^. Over the ensuing decades, human YFV infections have been acquired only in the forest cycle, despite the reinvasion of *Ae. aegypti* in Brazil from the late 1960s onwards^10^. Today, the forest YFV cycle is still very active in Brazil, and generates outbreaks every 6–10 years in the Southern, Southeast and Central-West regions, and every 14 years, in the Amazon^8, 11^. Humans are contaminated by the bite of forest canopy-dwelling mosquitoes of the genera *Haemagogus* (primary vectors) and *Sabethes* (secondary vectors)^4^.

**Figure 1:**
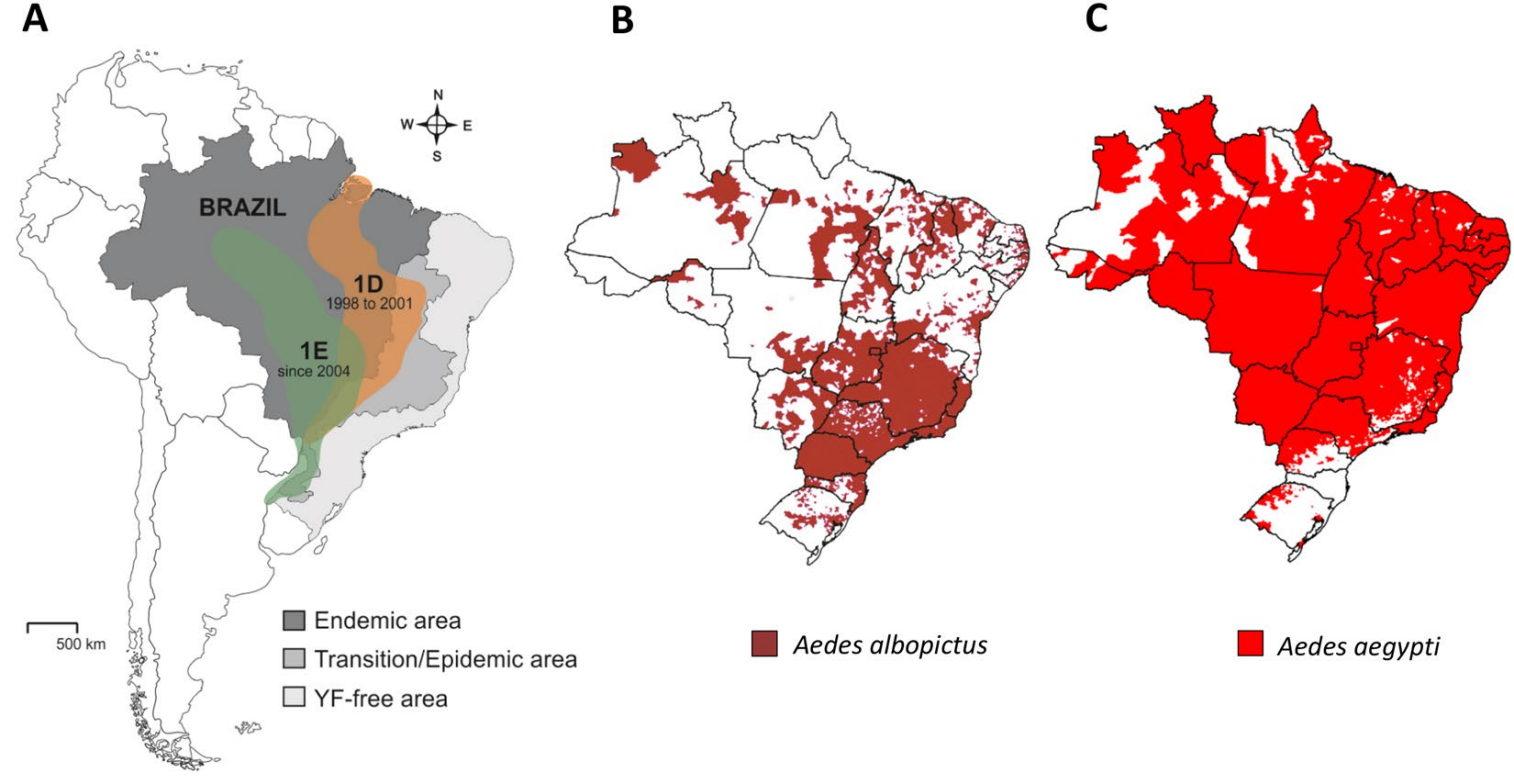
Geographic distribution of YFV strains (71016-1D and 4408-1E) (**A**), the mosquitoes *Ae. aegypti* (**B**) and *Ae. albopictus* (**C**) according to ref. 13. The map was created using software the CorelDraw X5 software (http://www.coreldraw.com/br/).

Since the late 1990s, YFV has extended its traditional range of distribution reaching southern and southeastern regions in Brazil, approaching the most densely populated and highly *Aedes-*infested cities having low vaccination coverage^12–14^. Thus, the YFV lineage belonging to the South America genotype I (named 1D) responsible for epizootics from 1998 to 2001^4^ has been replaced by a new viral lineage (1E) causing massive deaths of howler monkeys (*Alouatta caraya*) and human cases (Fig. 1)^15^. Since late 2016, a severe YFV epidemic has been reported in southeastern Brazil, causing 79 laboratory confirmed deaths. Most alarmingly, the ongoing epidemic has been progressively spreading toward the Atlantic coast, causing deaths of NHPs and humans in a zone free of YF for more than 70 years but highly infested by *Ae. aegypti* and *Ae. albopictus* (Brazilian Ministry of Health, “Ministério da Saúde divulga novos dados de febre amarela”, 2017; http://portalsaude.saude.gov.br/index.php/cidadao/principal/agencia-saude/27482-ministerio-da-saude-divulga-novos-dados-de-febre-amarela). If the re-establishment of *Ae. aegypti* posed a threat by itself^16^, the invasion and spread of *Ae. albopictus* since the late 1980s have stepped up the risk of urban YFV outbreaks in Brazil (Fig. 1)^13^. Moreover, some Brazilian populations of both *Ae. aegypti* and *Ae. albopictus* were susceptible to YFV^17–19^.

The mosquito *Ae. albopictus* is a very opportunistic species able to colonize a wide range of habitats besides being willing to feed on different mammals. It may also move from the forest to peri-urban sites and vice-versa^20, 21^. Coincidentally, the highest infestation indexes for *Ae. albopictus* in Brazil are reported in the Southeastern and Southern regions where YFV is now circulating^13^. We then hypothesize that YFV-competent *Ae. albopictus* may play the role of “bridge vector” linking the forest cycle to the urban YFV cycle. To address the question of the potential role of *Ae. albopictus* in the urban expansion of zoonotic YFV, we compare the vector competence for three YFV isolates of Brazilian and African populations of *Ae. albopictus* as well as of co-occurring populations of *Ae. aegypti*, and Neotropical sylvatic vectors from genera *Haemagogus* and *Sabethes*.

## Results

### *Aedes* mosquitoes were efficient to disseminate and transmit from day 14 post-infection

**Figure 2:**
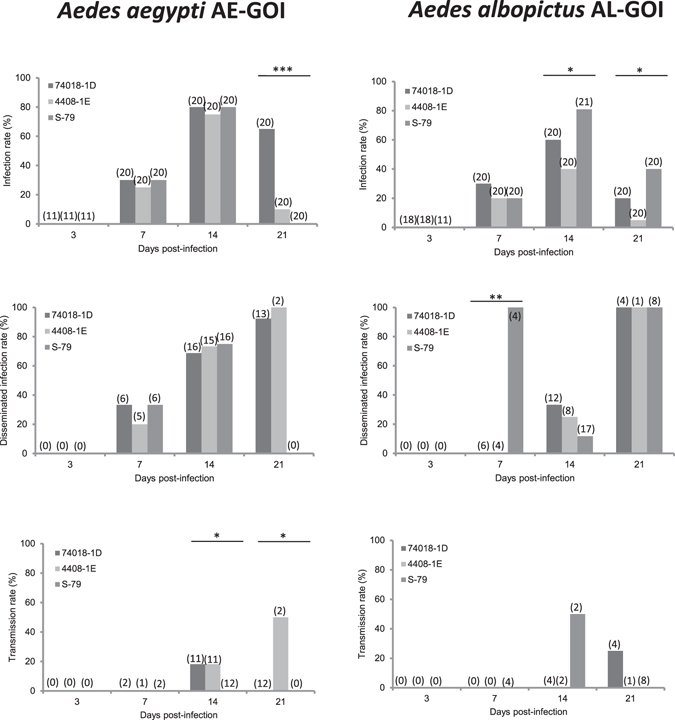
Infection, Dissemination and Transmission of YFV by *Aedes aegypti* AA-GOI and *Aedes albopictus* AL-GOI from the epizootic/epidemic region of Goiânia. Mosquitoes were exposed to blood meals at a titer of 10^6^ PFU/mL. Engorged females were maintained in laboratory conditions until examination at 3, 7, 14, 21 days post-infection. Mosquito thorax and abdomen were processed individually to determine the infection rate (IR, proportion of mosquitoes with infected body among the engorged mosquitoes). The mosquito head was used to define the disseminated infection rate (DIR, proportion of mosquitoes with infected head among infected mosquitoes) and the saliva collected from individual females to determine the transmission rate (TR, proportion of mosquitoes with infectious saliva among mosquitoes with disseminated infection). Asterisks refer to a significant difference (*p < 0.05, **p < 10^−2^, ***p < 10^−3^). In brackets, the number of mosquitoes tested.

To define the days after infection for examining viral dissemination and transmission in *Ae. aegypti* and *Ae. albopictus*, we infected *Ae. aegypti* AE-GOI and *Ae. albopictus* AL-GOI collected in Goiânia in the state of Goiás, Central Brazil, in the YFV-epidemic/epizootic region with three YFV strains (two from Brazil (74018-1D and 4408-1E) and one from Senegal (S-79)). When examining AE-GOI, mosquitoes were infected and disseminate YFV from 7 days post-infection (dpi), and viral particles were only detected in mosquito saliva at 14 dpi (Fig. 2). For AL-GOI, infection and dissemination started from 7 dpi and detection of virus in saliva from 14 dpi (Fig. 2).

Interestingly, out of 447 female mosquitoes studied at 3 dpi, no mosquitoes showed infection. Therefore, specimens examined at 3 dpi were not considered in further analysis. Moreover, considering all *Ae. aegypti* populations on the one hand, and all *Ae. albopictus* populations on the other hand, logistic regression models showed similar rates of infection at 14 and 21 dpi (p = 0.10 and p = 0.73, respectively). Therefore, all further analyses were conducted considering 14 and 21 dpi together.

### Brazilian *Aedes aegypti* were susceptible to American as well as African YFV genotypes

**Figure 3:**
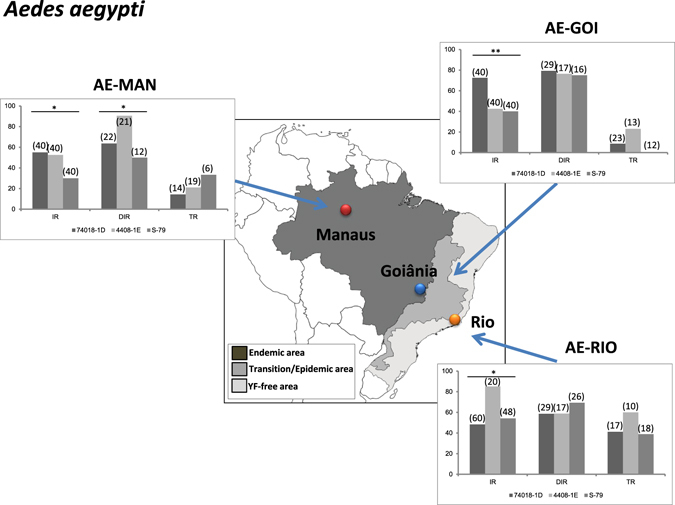
Vector competence of three *Aedes aegypti* populations (AA-MAN, AA-GOI and AA-RIO) for three YFV strains (74018-1D, 4408-1E and S-79). Mosquitoes were exposed to blood meals at a titer of 10^6^ PFU/mL. Engorged females were maintained in laboratory conditions until days 14–21 post-infection. Mosquitoes were processed as previously described to determine the infection rate (IR), the disseminated infection rate (DIR) and the transmission rate (TR). Asterisks refer to a significant difference (*p < 0.05, **p < 10^−2^). In brackets, the number of mosquitoes tested. The map was created using software the CorelDraw X5 software (http://www.coreldraw.com/br/).

To determine if *Ae. aegypti* populations from Brazil were competent vectors of YFV, three populations (AE-MAN, AE-GOI, AE-RIO collected in enzootic-, epizootic/epidemic- and free-YFV areas, respectively; Fig. 3) were experimentally infected with three YFV strains (74018-1D, 4408-1E and S-79). When analyzing viral infection, AE-MAN, AE-GOI and AE-RIO presented significant differences of IR for the three YFV strains (p < 0.05, Fig. 3) with values ranging from 30% (AE-MAN infected with S-79) to 85% (AE-RIO infected with 4408-1E). After controlling for population, virus and dpi, level of infection was higher with the strain 74018-1D in AE-GOI, was higher with the strain 4408-1E and 74018-1D in AE-MAN, while no difference was observed in AE-RIO (Table 1).

**Table 1:** Comparison of infection rates (logistic regression model*).

Population	Virus	Day post-infection	Species	
			*Aedes aegypti* (AE)	*Aedes albopictus* (AL)
Goiânia (GOI)	4408-1E	7	1	0.46 (0.26–0.83)
		14–21	2.58 (1.58–4.22)	1.19 (0.56–2.52)
	74018-1D	7	2.10 (1.13–3.91)	1.20 (0.59–2.42)
		14–21	5.43 (2.41–12.23)	3.10 (1.30–7.37)
	S-79	7	1.19 (0.64–2.21)	1.34 (0.66–2.70)
		14–21	3.08 (1.39–6.85)	3.45 (1.46–8.17)
Manaus (MAN)	4408-1E	7	2.87 (1.26–6.50)	0.32 (0.12–0.88)
		14–21	3.85 (1.79–8.24)	0.43 (0.17–1.08)
	74018-1D	7	2.75 (1.16–6.54)	0.38 (0.15–0.97)
		14–21	3.68 (1.67–8.09)	0.51 (0.23–1.12)
	S-79	7	1.41 (0.58–3.33)	0.38 (0.15–0.98)
		14–21	1.89 (0.84–4.22)	0.51 (0.22–1.19)
Rio de Janeiro (RIO)	4408-1E	7	5.90 (2.49–13.99)	0.28 (0.10–0.78)
		14–21	7.47 (3.20–17.44)	0.36 (0.13–0.95)
	74018-1D	7	3.28 (1.41–7.63)	0.19 (0.07–0.53)
		14–21	4.15 (1.96–8.79)	0.29 (0.10–0.61)
	S-79	7	3.64 (1.56–8.49)	0.42 (0.17–1.08)
		14–21	4.61 (2.13–9.98)	0.53 (0.23–1.26)
Congo (CON)	4408-1E	7	2.82 (1.27–6.25)	1.57 (0.67–3.82)
		14–21	2.44 (1.18–5.06)	1.39 (0.61–3.17)
	74018-1D	7	1.36 (0.55–3.39)	0.96 (0.41–2.23)
		14–21	1.18 (0.51–2.71)	0.83 (0.38–1.84)
	S-79	7	1.59 (0.65–3.91)	2.20 (0.99–4.88)
		14–21	1.38 (0.61–3.12)	1.91 (0.91–2.98)

*Model with interaction between species and population, between species and virus, between population and virus, and also between population and day post-infection.

After midgut infection, the virus must propagate inside the mosquito hemocele to allow detecting a positive viral dissemination. Dissemination as determined by the presence of virus in mosquito heads, were similar regardless of the YFV strain except for AE*-*MAN which presented higher viral dissemination of YFV 4408-1E (90.47%) (Fig. 3, Table 2).

**Table 2:** Comparison of disseminated infection rates (logistic regression model*).

Population	Virus	Species	
		*Aedes aegypti* (AE)	*Aedes albopictus* (AL)
		Adj. OR [95% CI]	Adj. OR [95% CI]
Goiânia (GOI)	4408-1E	1	0.28 (0.14–0.58)
	74018-1D	1.49 (0.60–3.70)	0.42 (0.14–1.31)
	S-79	1.64 (0.64–4.18)	0.46 (0.16–1.33)
Manaus (MAN)	4408-1E	3.76 (1.16–12.17)	6.84 (1.38–33.81)
	74018-1D	1.03 (0.37–2.86)	1.88 (0.46–7.69)
	S-79	1.00 (0.31–3.22)	1.82 (0.48–6.95)
Rio de Janeiro (RIO)	4408-1E	1.43 (0.48–4.27)	1.36 (0.35–5.32)
	74018-1D	0.93 (0.35–2.45)	0.88 (0.23–3.42)
	S-79	1.08 (0.41–2.86)	1.03 (0.26–4.13)
Congo (CON)	4408-1E	6.30 (1.85–21.46)	5.31 (1.47–19.16)
	74018-1D	3.94 (0.93–16.65)	3.31 (0.85–12.95)
	S-79	1.12 (0.36–3.47)	0.94 (0.33–2.66)

*Adjusted for time, with interaction between species and population and also between population and viral strain.

For viral transmission to occur, the virus in the hemocele must reach mosquito salivary glands and be excreted with saliva expectorated by the mosquito. TR as determined by the presence of virus in saliva, were similar for all three Brazilian *Ae. aegypti* populations regardless of the YFV strain (p > 0.05; Fig. 3, Table 3). These results suggest that the salivary glands behave as a more efficient barrier for the release of viruses than the midgut for dissemination. Moreover, all mosquito populations present similar ability to deliver particles of the two YFV strains in saliva: 74018-1D responsible for epizootics from 1998 to 2001^4^ and the new viral lineage, 4408-1E, causing increasing deaths of monkeys and human cases^15^.

**Table 3:** Comparison of transmission rates (logistic regression model*).

	Adjusted OR (95% CI)	P
**Species**		
*Aedes aegypti* (AE)	1	0.95
*Aedes albopictus* (AL)	0.98 (0.52–1.83)	
**Population**		
Congo (CON)	6.97 (2.75–17.65)	<0.001
Goiânia (GOI)	1	
Manaus (MAN)	2.63 (0.97–7.10)	
Rio de Janeiro (RIO)	7.93 (3.08–20.39)	
**Virus**		
4408-1E	1	0.14
74018-1D	0.70 (0.36–1.38)	
S-79	0.49 (0.24–1.00)	
**Day post-infection**		
7	1	<0.001
14–21	13.98 (4.12–47.48)	

*No significant interaction.

### *Aedes albopictus* in Rio de Janeiro were very efficient to deliver particles of YFV from their saliva

**Figure 4:**
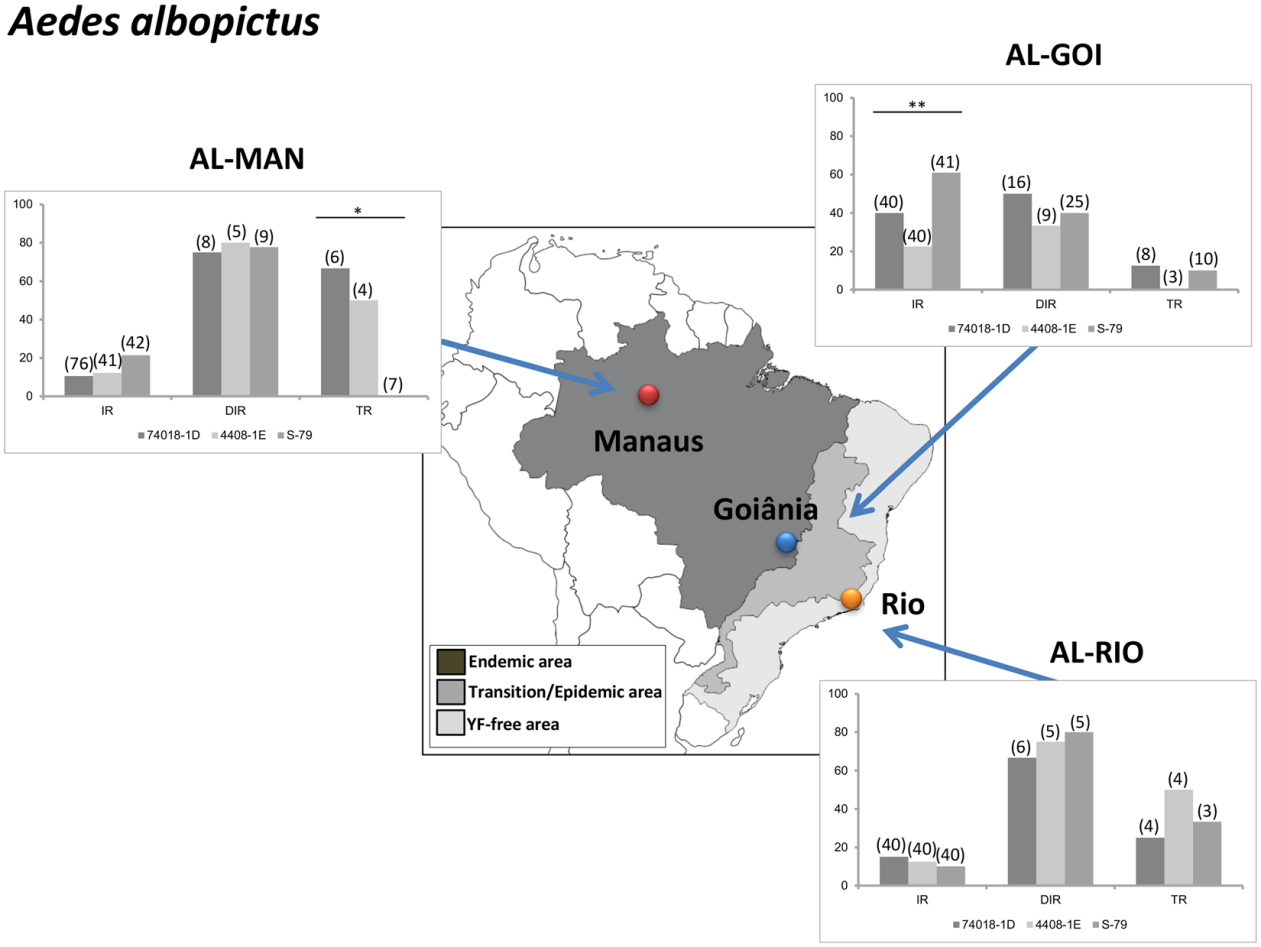
Vector competence of three *Aedes albopictus* populations (MAA, GOA and PMNI) for three YFV strains (74018-1D, 4408-1E and S-79). Mosquitoes were exposed to blood meals at a titer of 10^6^ PFU/mL. Engorged females were maintained in laboratory conditions until days 14–21 post-infection. Mosquitoes were processed as previously described to calculate the infection rate (IR), the disseminated infection rate (DIR) and the transmission rate (TR). Asterisks refer to a significant difference (*p < 0.05, **p < 10^−2^). In brackets, the number of mosquitoes tested. The map was created using software the CorelDraw X5 software (http://www.coreldraw.com/br/).

To determine if *Ae. albopictus* populations from Brazil were as competent vectors as *Ae. aegypti* for YFV, *Ae. albopictus* from Manaus (AL-MAN), Goiânia (AL-GOI) and Rio (AL-RIO) were experimentally infected with three YFV strains (two from Brazil and one from Senegal). When comparing viral infection, AL-MAN and AL-RIO presented low (ranging from 10% for AL-RIO infected with S-79 to 21.42% for AL-MAN infected with S-79; p > 0.05; Fig. 4), and comparable IR values whatever the viral strain (p = 0.71 and p = 0.19, respectively; Table 1). On the other hand, AL-GOI showed significant differences (p < 0.05) with a higher IR value for S-79 (60.97%) (Table 1). It must also be noted that the rate of infection was significantly lower in *Ae. albopictus* than in *Ae. aegypti* for all viral strains, except in females from Goiânia when infected with S79.

When analyzing viral dissemination represented by DIR, dissemination was lower in AL-GOI, then higher in AL-RIO and then even higher in AL-MAN (Fig. 4, Table 2). All three *Ae. albopictus* populations showed similar values (p > 0.05) for the three YFV, although for AL-MAN, dissemination tended to be higher with viral strain 1E (Table 2). The highest DIR values were obtained for AL-MAN and AL-RIO populations. Interestingly, dissemination was similar in AL and in AE from Manaus and from Rio; while in female mosquitoes from GOI, the dissemination was significantly lower in AL than in AE (Table 2).

When considering viral transmission described by TR, AL-MAN population showed high TR values but based on low sample sizes (66.67% (6) with 74018-1D and 50% (4) with 4408-1E) and surprisingly, was not able to transmit the S-79 YFV from Africa. The population AL-GOI collected in the epizootic/epidemic region were able to excrete similarly all three YFV (p > 0.05) but was less efficient than AL-RIO (Fig. 4). These results suggest that AL-RIO and AL-MAN shared the same pattern of infection, dissemination and transmission with low IR, high DIR and high TR values suggesting the role of the midgut as the main barrier in the trajectory of the virus to the mosquito salivary glands. Interestingly, AL-GOI was less susceptible to YFV than the other two mosquito populations. Like *Ae. aegypti*, *Ae. albopictus* were similarly susceptible to the former YFV 74018-1D lineage and the new viral lineage 4408-1E (Table 3).

### *Aedes* mosquitoes from an African YFV-endemic country were similarly susceptible to American as well as African YFV genotypes

To test whether *Ae. aegypti* and *Ae. albopictus* from Congo were competent for both Brazilian and West African YFV strains, mosquitoes were infected with the three YFV strains. The population AE-CON and AL-CON showed IR ranging between 25% (when infected with 74018-1D) and 38.6% (when infected with 4408-1E). Whatever the viral strain, the infection rate was not different between AE-CON and AL-CON (p > 0.05; Table 1)

**Figure 5:**
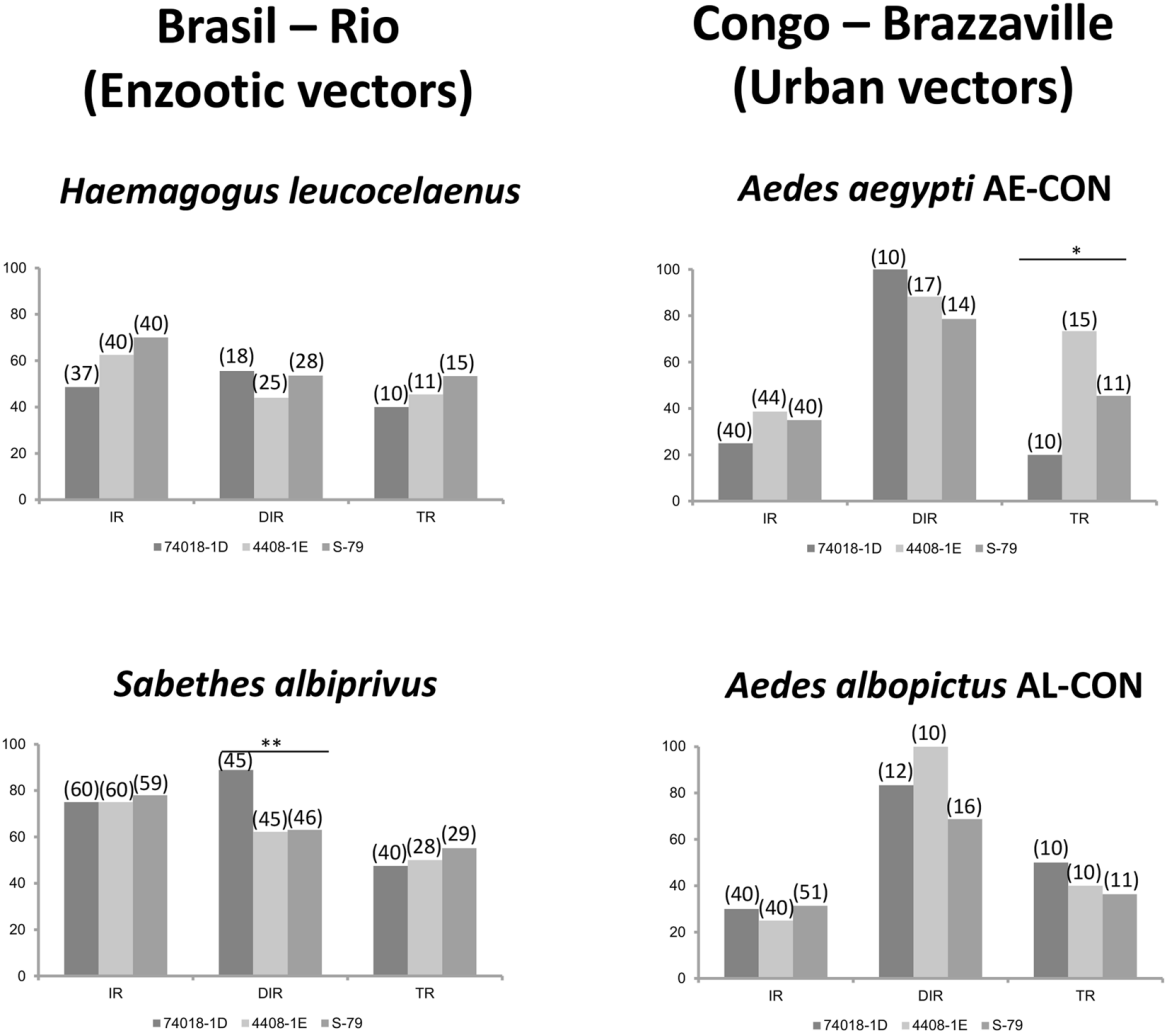
Vector competence to YFV of Brazilian enzootic vectors (*Haemagogus leucocelaenus* and *Sabethes albiprivus*) and Congolese domestic vectors (*Aedes aegypti* and *Aedes albopictus*) used as controls of YFV infection. Mosquitoes were on an infectious blood meal provided at a titer of 10^6^ PFU/mL. Mosquitoes were processed as previously described. IR indicates to the infection rate, DIR to the disseminated infection rate and TR to the transmission rate. Asterisks refer to a significant difference (*p < 0.05, **p < 10^−2^). In brackets, the number of mosquitoes tested.

Regarding dissemination, AE-CON and AL-CON also showed much higher viral dissemination rates than the other female mosquitoes (Fig. 5). Interestingly, American viral strains led to significantly higher dissemination than the African one both for AE-CON and for AL-CON (p < 0.05), but we did not observe any difference between AE-CON and AL-CON (p = 0.72).

Viral transmission measured by TR was slightly lower except when infected with the YFV 4408-1E strain (73.33%). On the other hand, AL-CON presented roughly similar patterns than AE*-*CON with IR lower than 31.37% (S-79) and DIR higher than 68.75% (S-79). TR did not differ significantly (p > 0.05), varying from 36.36% (S-79) to 50% (74018-1D) strains. Thus *Ae. aegypti* and *Ae. albopictus* from Congo, city of Brazzaville presented similar vector competence indices when infected with YFV strains belonging to both the South America I and West Africa genotypes.

### Wild YFV vectors *Hemagogus* and *Sabethes* from Rio de Janeiro were highly competent to transmit Brazilian and African YFV strains

To examine if enzootic mosquitoes from Brazil, *Hg. leucocelaenus* and *Sa. albiprivus* were as susceptible as *Ae. aegypti* and *Ae. albopictus*, mosquitoes were infected with the three YFV strains. Both enzootic mosquito species showed pattern of infection similar to *Ae. aegypti* while *Ae. albopictus* was significantly less often infected (as previously shown in this study). Overall, dissemination occurred in 64.3% of mosquitoes, this rate of dissemination was not different between the four species (p = 0.34), between the three viruses (p = 0.14). Overall, transmission was observed in 36.6% and was not different between the four species (p = 0.85) nor between the three viruses (p = 0.95). IRs were higher than 48%, DIRs higher than 44% and TRs higher than 40% suggesting a limited role of both barriers, midgut and salivary glands, in the virus migratory route inside the mosquito. When mosquitoes with infectious saliva were considered according to the initial number of females tested, transmission efficiencies (TE) varied from 10.81% to 20% for *Hg. leucocelaenus* and 23.33% to 31.66% for *Sa. albiprivus* (Supplementary Figure). Thus enzootic YFV vectors from Brazil were highly competent to transmit YFV from Brazil as well as from West Africa.

## Discussion

Our results showed that *Ae. aegypti* and *Ae. albopictus* from YFV-free regions in Brazil were as susceptible as their counterparts in the endemic Amazon region to transmit YFV. Mosquitoes from the epizootic/epidemic region were broadly less competent suggesting that *Ae. albopictus* and to a lesser extent, *Ae. aegypti*, were unlikely to play the role of active bridge vector in the emergence zone even if it cannot definitively be excluded.

Since the main YF-vector *Ae. aegypti* has been eradicated from Brazil in 1957^9^, YFV had been maintained within a forest cycle in endemic/enzootic regions where waves of epizootics coincided with the renewal of non-human primate populations, taking an interval of 6–10 years^22^. Consequently to the reintroduction of *Ae. aegypti* and the new establishment of *Ae. albopictus* in the 1970–80 s, YFV distribution has extended to the Central-West of Brazil (state of Mato Grosso), the Northeast (state of Bahia), Southeast (states of Minas Gerais, Espírito Santo, and São Paulo), and the Southern^23^. In 2008–2009, an YFV outbreak hit Rio Grande do Sul^24^ and São Paulo^15^ caused by a new lineage 1E replacing the former 1D^4^. We showed that Brazilian *Ae. aegypti* and *Ae. albopictus* as well as wild vectors (*Haemagogus leucocelaenus* and *Sabethes albiprivus*) were equally susceptible to the two lineages ruling out differences of vector competence as a cause of viral replacement.

Enzootic mosquitoes *Hg. leucocelaenus* was the most dominant species in forests close to Rio de Janeiro beside *Ae. albopictus* ^25^. We showed that *Hg. leucocelaneus* as well as *Sa. albiprivus* were highly susceptible to all three YFV strains corroborating their role as YFV-enzootic vectors^26^. They were able to experimentally deliver infectious viral particles in saliva after infection with the two YFV strains, the former 1D and the new viral lineage 1E, as well as the West African YFV strain isolated from Senegal in 1979. Their pattern of YFV infection, dissemination and transmission are roughly similar to those of domestic *Ae. aegypti* and *Ae. albopictus* from Brazzaville in Congo acting as potential urban vectors in Central Africa^27^. YFV is at the gates of the most densely populated zone in South America, the Brazilian Atlantic coast, where are located large cities like Rio de Janeiro (WHO, “Yellow fever – Brazil”, 2017; http://www.who.int/csr/don/27-january-2017-yellow-fever-brazil/en/). *Ae. albopictus* colonizes regions surrounding the urban environment where *Ae. aegypti* remains the main vector of arboviruses pathogen to humans (dengue, chikungunya, Zika). As expected, we showed that both mosquito species were able to excrete YFV from day 14^28^. Populations of *Ae. aegypti* and *Ae. albopictus* from -endemic, -epizootic/epidemic, and YFV-free regions were compared for their performance to be infected, disseminate and transmit YFV. We brought out that mosquitoes from the YFV-endemic (i.e. Manaus) and YFV-free regions (i.e. Rio de Janeiro) were more capable after oral infections to expectorate the two American (74018-1D and 4408-1E) and one African (S-79) YFV strains than mosquitoes from the epizootic/epidemic area (i.e. Goiânia). These two geographically distant regions are linked by an emergence region where mosquitoes are susceptible to YFV infection. *Ae. albopictus* as well as *Ae. aegypti* from Goiânia are able to deliver the three YFV strains in their saliva after experimental infections but with much less efficiency. However, a poorly competent vector may play an important role in transmission if other conditions are met, e.g. high vector densities, high human-biting rate and high daily survival rates^29^. Moreover, it has been shown that human population has extensively contributed to YFV dispersal^2^. Some environmental/ecological barriers by inhibiting movements of vectors and hosts can prevent YFV spillovers from a forest cycle. However, these barriers became more and more anecdotal in Brazil as with population growth, cities are increasingly close to YFV-enzootic forests. Besides, because of their original habitat degradation and their high propensity to explore new environments, NHPs such as capuchins and marmosets have densely invaded parks and urban areas in Rio de Janeiro and other cities in the Atlantic coast. These new urban invaders colonize a large area around cities including patches of forests^30, 31^ where sylvatic YFV vectors can be abundant^32^. Considering all these conditions, it is difficult to understand why YF is not already in the urban areas of Brazil. However, it seems that this is changing with the recent detection of human cases at less than that 100 km apart from city of Rio de Janeiro (http://portalarquivos.saude.gov.br/images/pdf/2017/abril/24/COES-FEBRE-AMARELA-INFORME-37.pdf).

The emergence in Angola in January 2016 exemplifies the threat of YFV spreading outside its historical cradle. Starting from Luanda where YFV caused nearly 600 deaths, it reached neighboring countries, the Democratic Republic of Congo (DRC) in March 2016 and Uganda in April 2016^32^. Most cases were found in cities suggesting that transmission implicates urban vectors, mainly *Ae. aegypti*. Imported cases from Angola were later confirmed in China^33^ stressing the risk of spread outside Africa in *Aedes*-infested countries through non-immunized travelers. All four mosquito species, *Ae. aegypti*, *Ae. albopictus*, *Hg. leucocelaenus* and *Sa. albiprivus* from Rio de Janeiro were highly susceptible to Brazilian as well as West African YFV lineages. The city of Rio de Janeiro is an important touristic and trade center hosting nearly 6.5 million inhabitants, and where annually converge 26 millions of Brazilians/tourists in airports and 19 million via roads (http://cidades.ibge.gov.br/xtras/perfil.php?codmun=330455). If the virus is introduced from an infected traveler into Rio de Janeiro, opportunities to initiate a vectorial transmission of YFV are multiple: (i) by anthropophilic mosquitoes such as *Ae. aegypti* and *Ae. albopictus* which are highly susceptible to YFV and (ii) by YFV-enzootic mosquitoes *Hg. leucocelaenus* and *Sa. albiprivus* colonizing the forest near Rio de Janeiro. Therefore, vaccination of travelers visiting Rio de Janeiro should be highly advised to limit the risk of introducing the virus from YFV-endemic areas.

An effective YFV vaccine has been available since the 1930s. Unfortunately, incomplete coverage in regions at risk of infection is responsible for several thousands of deaths every year. In the Americas, to prevent enzootic spillovers with introduction of YFV into the urban cycle, people in contact with the jungle should be rapidly vaccinated in priority to prevent a potential urbanization of YFV.

## Methods

### Ethics Statements

The Institut Pasteur animal facility has received accreditation from the French Ministry of Agriculture to perform experiments on live animals in compliance with the French and European regulations on care and protection of laboratory animals. This study was approved by the Institutional Animal Care and Use Committee (IACUC) at the Institut Pasteur and by the Institutional Ethics Committee on Animal Use (CEUA-IOC license LW-34/14) at the Instituto Oswaldo Cruz. Mosquito collections in the Atlantic forest in Rio de Janeiro were approved by local environmental authorities (PNMNI license 001/14-15; SISBIO-MMA licenses 37362-2 and 012/2016). No specific permits were required for performing mosquito collection in the urban and suburban areas in Brazil and Congo. This study did not involve endangered or protected species.

### Mosquitoes

**Figure 6:**
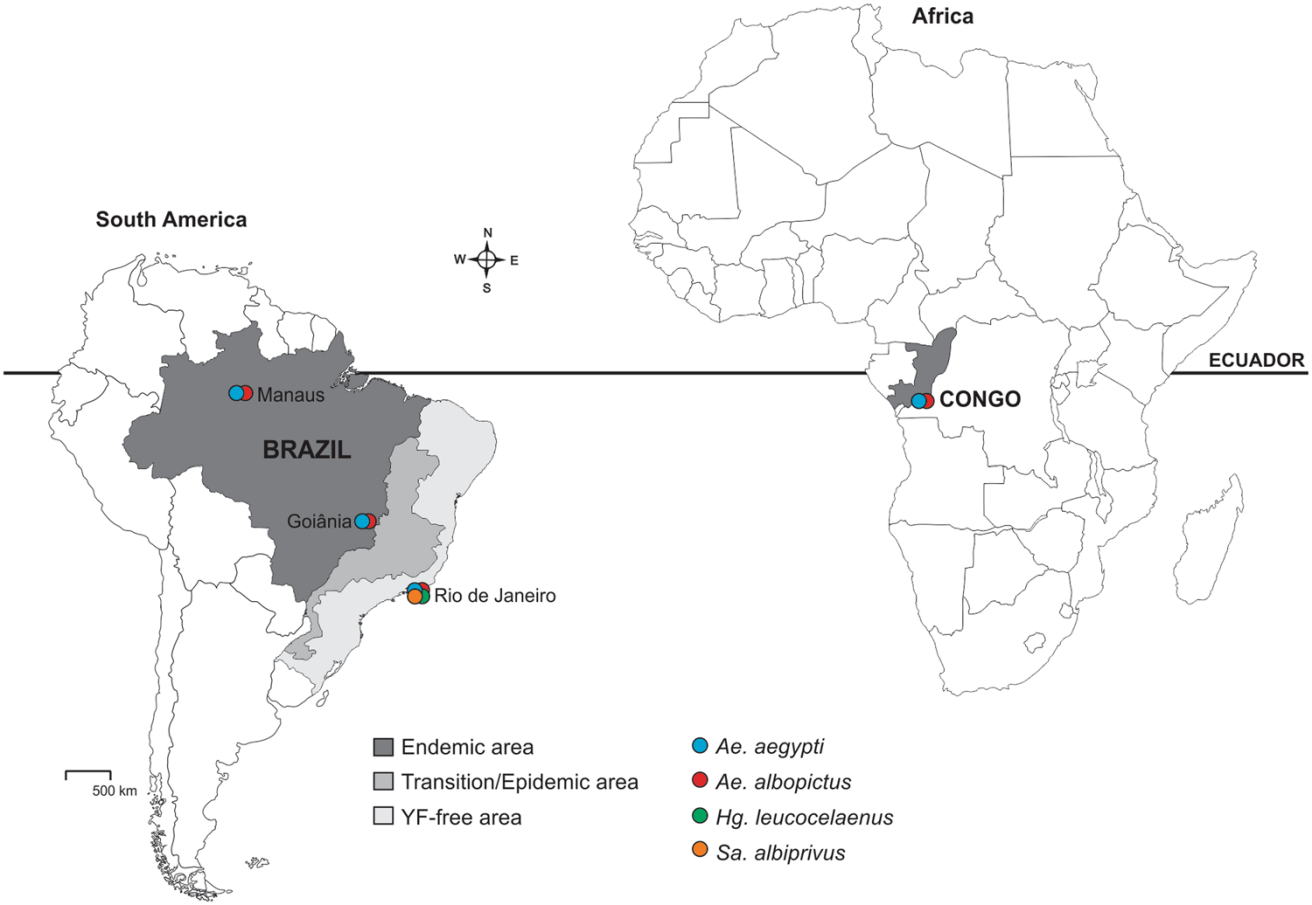
Geographical localization of tested mosquitoes in Brazil and Congo. The map was created using software the CorelDraw X5 software (http://www.coreldraw.com/br/).

Ten American and African mosquito populations originated from three contrasting regions (enzootic, epidemic/epizootic and YFV-free areas) were challenged with YFV: four *Ae. albopictus*, four *Ae. aegypti*, one *Haemagogus leucocelaenus* (Dyar & Shannon) and one *Sabethes albiprivus* Theobald (Fig. 6, Table 4). We tested paired *Ae. albopictus* and *Ae. aegypti* populations simultaneously sampled in the same area (i.e. Brazil and Congo). Similarly, four species (*Ae. albopictus, Ae. aegypti*, *Hg. leucocelaenus* and *Sa. albiprivus*) collected in the Rio de Janeiro area were tested (Table 1). Populations were derived from eggs collected with ovitraps settled on the ground for sampling *Ae. albopictus* and *Ae. aegypti* in the urban and suburban sites, or suspended at the forest canopy at 5–16 m high for collecting *Hg. leucocelaenus*. When possible, the first generation in laboratory after collection (F1) of *Ae. aegypti* and *Ae. albopictus* was used for experimental infections. For *Hg. leucocelaenus* which cannot be maintained in laboratory conditions^34, 35^, the F0 generation derived from eggs collected fortnightly in 2015 was directly used for infections. In the case of *Sa. albiprivus*, mosquitoes from a colony established in the laboratory since 2013 were used.

**Table 4:** Mosquito populations challenged with YFV.

Mosquito Population	Collection site	Continent	Country	Epidemiological scenario	Generation used	Mosquito Specie
AL-MAN	Manaus, Amazônia	South America	Brazil	Endemic	F_1_	*Ae. albopictus*
AE-MAN	Manaus, Amazônia	South America	Brazil	Endemic	F_1_	*Ae. aegypti*
AL-GOI	Goiânia, Goiás	South America	Brazil	Epizootic/epidemic	F_1_	*Ae. albopictus*
AE-GOI	Goiânia, Goiás	South America	Brazil	Epizootic/epidemic	F_1_	*Ae. aegypti*
AL-RIO	Nova Iguaçu, Rio de Janeiro	South America	Brazil	YF-free area	F_1_	*Ae. albopictus*
AE-RIO	Urca, Rio de Janeiro	South America	Brazil	YF- free area	F_1_	*Ae. aegypti*
Haemagogus	Nova Iguaçu, Rio de Janeiro	South America	Brazil	YF-free area	F_0_	*Hg. leucocelaenus*
Sabethes	Tinguá, Rio de Janeiro	South America	Brazil	YF-free area	>F_10_	*Sa. albiprivus*
AL-CON	ORSTOM campus, Brazzaville	Africa	Congo	Epizootic/Endemic	>F_10_	*Ae. albopictus*
AE-CON	ORSTOM campus, Brazzaville	Africa	Congo	Epizootic/Endemic	>F_10_	*Ae. aegypti*

Ovitraps were provided with 1 to 3 wooden paddles. Eggs were hatched by submerging paddles in dechlorinated tap water for two consecutive days. Larvae were reared in pans (25 × 25 × 10 cm) containing one liter of dechlorinated tap water, supplemented with yeast powder renewed every 2–3 days. In the case of *Hg. leucocelaenus*, we added senesced leaves besides yeast powder in water. We reared ~100 larvae/pan for *Ae. albopictus* and *Ae. aegypti*, and ~50 larvae/pan for *Hg. leucocelaenus* and *Sa. albiprivus*. The emerged F_0_ adults were morphologically identified^36^ maintained in insectaries (27 ± 1 °C; 80 ± 10% RH; 16 h:8 h light:dark cycle) and supplied with both 30% sucrose and/or honey solutions. *Ae. albopictus, Ae. aegypti*, and *Sa. albiprivus* females were fed three times a week on anesthetized mice to produce eggs.

### Viruses

Mosquitoes were challenged with three YFV isolates: two belonging to the South America genotype I, isolated from Brazil, corresponding to two distinct lineages FIOCRUZ 74018/MG/01 (YFV-74018), isolated from a human fatal case in 2001, belonging to the lineage 1D^37^, and IEC-4408 (YFV-4408), isolated from a howler-monkey in 2008, from the lineage 1E (GenBank KY861728), and one strain [S79-P4 (YFV-S79)] from the West African lineage, isolated from a human case in Senegal in 1979^38^. YFV-74018 and YFV-4408 were isolated from serum in *Ae. albopictus* C6/36 cell culture, and passaged four times on the same cell line, while YFV-S79 was passaged twice on newborn mice and two times on C6/36 *Ae. albopictus* cells. Viral stocks of all strains were produced on C6/36 *Ae. albopictus* cells and stored at −80 °C until used for the mosquito experimental infection assays.

### Mosquito experimental assays

Six- to 8-day-old females were grouped in feeding boxes (60 females/box) and starved for 24 h, except for *Sa. albiprivus* which were starved for 48 h. Females were fed on an infectious blood meal containing two parts of washed rabbit erythrocytes, one part of the viral suspension supplemented with a phagostimulant (ATP) at a final concentration of 5 mM maintained at 37 °C. The infectious blood-meals contained a final viral titer of 10^6^ PFU/mL. Mosquito feeding period was limited to 1 hour. Only fully engorged females were incubated at 28 °C constant temperature, 80% RH and 16 h:8 h light:dark cycle, with daily access to 10% sucrose or honey solution. For each combination mosquito population-YFV strain, samples of 20 mosquitoes were examined at 3, 7, 14 and 21 days after virus exposure for determining vector competence indices. Infection rate (IR) refers to the proportion of mosquitoes with infected body among the engorged ones. Disseminated infection rate (DIR) corresponds to the proportion of mosquitoes with infected head among the previously detected infected mosquitoes (i.e, abdomen/thorax positive). Transmission rate (TR) represents the proportion of mosquitoes with infectious saliva among mosquitoes with disseminated infection^39^. Even if this is an indication, TR should not be regarded as a measure of transmission from mosquitoes to a vertebrate host.

Mosquitoes were processed as follows: abdomen and thorax (herein after referred to as body) were tested for determining infection, head for dissemination and saliva for transmission. To determine viral infection and dissemination rates, each mosquito body and head were respectively ground in 500 μL and 300 μL of Leibovitz L15 medium (Invitrogen) supplemented with 2% fetal bovine serum (FBS), centrifuged at 10,000 × g for 5 min at +4 °C and inoculated onto monolayers of *Ae*. *albopictus* C6/36 cell culture in 96-well plates. After 1 h incubation at 28 °C, 150 μL of 2.4% CMC (carboxymethyl cellulose) in Leibovitz L15 medium supplemented with 10% FBS was added per well. After 5 days incubation at 28 °C, cells were fixed with 10% formaldehyde, washed, and revealed using hyperimmune ascetic fluid specific to YFV as the primary antibody and Alexa Fluor 488 goat anti-mouse IgG as the second antibody (Life Technologies)^28^. To estimate viral transmission, mosquito saliva was collected in individual pipette tips containing 5 μL FBS for 30 min as previously described^40^. FBS containing mosquito saliva was expelled into 45 μL of Leibovitz L15 medium, inoculated on *Ae*. *albopictus* C6/36 cell culture in 96-well plates and stained as described above.

### Statistical analysis

Rates (infection, disseminated infection, and transmission) were described using median and inter-quartile range (IQR). The effect of species, population, YFV strain and duration on the rates was investigated using logistic linear regression models. Two-by-two interaction between species, population, YFV strain and duration was systematically investigated. Statistical analyses were conducted using the Stata software (StataCorp LP, Texas, and USA). P-values < 0.05 were considered significant.

## Electronic supplementary material

Supplementary information
