# Supplementary material for: Potential risk of re-emergence of urban transmission of Yellow Fever virus in Brazil facilitated by competent Aedes populations
Source: Sci Rep. 2017 Jul 7;7:4848. doi: 10.1038/s41598-017-05186-3 (PMC5501812; doi:10.1038/s41598-017-05186-3)
Supplement: Supplementary file 1 — Supplementary information [file 41598_2017_5186_MOESM1_ESM.doc]

**Potential risk of re-emergence of urban transmission of Yellow Fever virus in Brazil facilitated by competent *Aedes* populations.** Dinair Couto-Lima,^1,2^ Yoann Madec,^3^ Maria Ignez Bersot,^1^ Stephanie Silva Campos,^1^ Monique de Albuquerque Motta,^1^ Flávia Barreto dos Santos,^1^ Marie Vazeille,^2^ Pedro Fernando da Costa Vasconcelos,^4^ Ricardo Lourenço-de-Oliveira,^1,¶^ Anna-Bella Failloux^2,¶^

**Supplementary Figure | Transmission efficiencies of *Ae. aegypti* populations (AE-MAN, AE-GOI and AE-RIO), *Ae. albopictus* populations (AL-MAN, AL-GOI and AL-RIO) and enzootic vectors (*Hg. leucocelaenus* and *Sa. albiprivus*) for three YFV strains (74018-1D, 4408-1E and S-79).** Mosquitoes were exposed to blood meals at a titer of 10^6^PFU/mL. Engorged females were maintained in laboratory conditions until 14-21 days post-infection. Mosquito saliva were collected and titrated on *Ae. albopictus* C6/36 cells. Transmission efficiency was calculated as the proportion of mosquitoes with infectious saliva among the initial number of mosquitoes used. In brackets, the number of mosquitoes tested. The map was created using software the CorelDraw X5 software (http://www.coreldraw.com/br/).

**
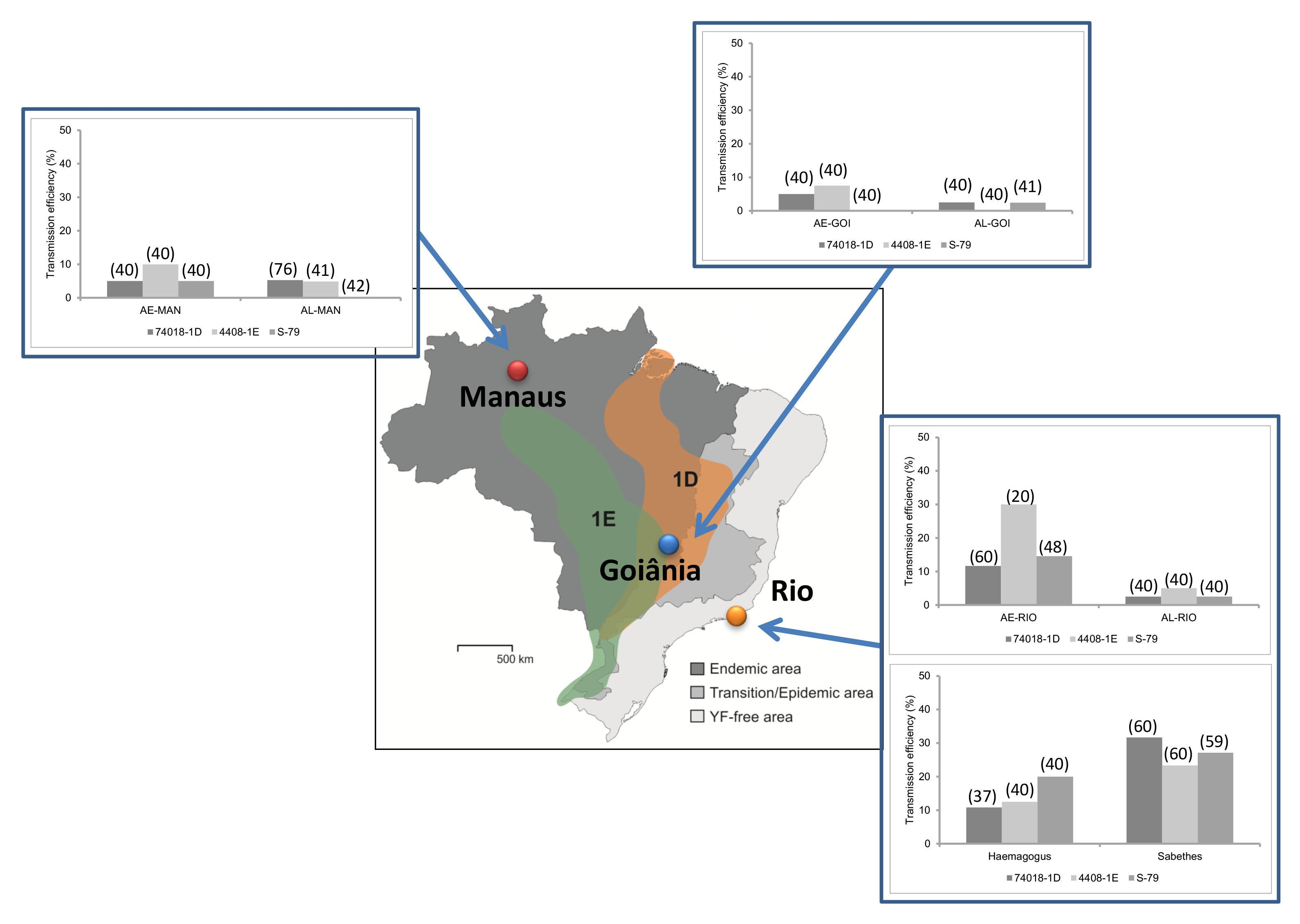
**
